# The mitochondrial ADP/ATP carrier exists and functions as a monomer

**DOI:** 10.1042/BST20190933

**Published:** 2020-07-29

**Authors:** Edmund R.S. Kunji, Jonathan J. Ruprecht

**Affiliations:** Medical Research Council Mitochondrial Biology Unit, University of Cambridge, Cambridge Biomedical Campus, Keith Peters Building, Hills Road, Cambridge CB2 0XY, U.K.

**Keywords:** adenine nucleotide translocase, biophysical techniques, molecular mass determination, native mass spectrometry, oligomeric state, structure

## Abstract

For more than 40 years, the oligomeric state of members of the mitochondrial carrier family (SLC25) has been the subject of debate. Initially, the consensus was that they were dimeric, based on the application of a large number of different techniques. However, the structures of the mitochondrial ADP/ATP carrier, a member of the family, clearly demonstrated that its structural fold is monomeric, lacking a conserved dimerisation interface. A re-evaluation of previously published data, with the advantage of hindsight, concluded that technical errors were at the basis of the earlier dimer claims. Here, we revisit this topic, as new claims for the existence of dimers of the bovine ADP/ATP carrier have emerged using native mass spectrometry of mitochondrial membrane vesicles. However, the measured mass does not agree with previously published values, and a large number of post-translational modifications are proposed to account for the difference. Contrarily, these modifications are not observed in electron density maps of the bovine carrier. If they were present, they would interfere with the structure and function of the carrier, including inhibitor and substrate binding. Furthermore, the reported mass does not account for three tightly bound cardiolipin molecules, which are consistently observed in other studies and are important stabilising factors for the transport mechanism. The monomeric carrier has all of the required properties for a functional transporter and undergoes large conformational changes that are incompatible with a stable dimerisation interface. Thus, our view that the native mitochondrial ADP/ATP carrier exists and functions as a monomer remains unaltered.

## Introduction

The determination of the oligomeric state of transport proteins is an important step in resolving their mechanism. The oligomeric state can be assessed by different sizing techniques, by binding and transport studies or by biophysical and structural methods. However, these studies are often marred with controversy. Since these experiments deal with membrane proteins, they are technically challenging, as many complicating factors often lead to incorrect assignments of the oligomeric state.

In this review, we discuss studies related to the oligomeric state of the mitochondrial ADP/ATP carrier, also called the adenine nucleotide translocator or translocase, which belongs to the mitochondrial carrier family (SLC25) [1–4]. Mitochondrial carriers form the largest solute carrier family in humans and are involved in the transport of amino acids, carboxylic acids, fatty acids, cofactors, inorganic ions and nucleotides across the mitochondrial inner membrane [1–4]. Their transport activities are central to many cellular processes, such as the oxidation of degradation products of fats and sugars, the synthesis of iron sulfur clusters and haem, and the degradation, synthesis and interconversion of amino acids, as well as mitochondrial macromolecular synthesis, ion homeostasis, heat production, mitochondrial dynamics, signalling, cellular differentiation, development and cell death [1–3,5]. Disease variants of mitochondrial carriers lead to a diverse range of neuromuscular, metabolic and developmental diseases [3,4].

The mitochondrial ADP/ATP carrier is the best-studied member of the SLC25 family. It was one of the first transporters to be characterised in detail, because of its natural abundance [6–8]. Based on a careful analysis of the binding properties of two specific inhibitors, carboxyatractyloside and bongkrekic acid, an alternating access mechanism was proposed, also called the single binding centre gated pore mechanism [6]. When the first amino acid sequences were determined [9–11], it became clear that mitochondrial carriers consist of three homologous repeats [12] and have six transmembrane α-helices [13]. Back then, it was thought that a six-helical membrane protein might be too small for transporting large substrates, such as ATP and palmitoyl carnitine. A dimer would be more compatible with the 12 α-helical topologies generally observed at the time and it was suggested that dimerisation might be a key generic property of transporters [14]. The results of a large number of biophysical and biochemical experiments subsequently seemed to support the notion that mitochondrial carriers exist and function as dimers (reviewed in [15]). However, when the first structural information became available, it became clear that the mitochondrial ADP/ATP carrier is a monomer with a three-fold pseudo-symmetrical structure and a translocation pathway through the centre, indicating that it could work as a monomer as well [16]. Subsequent atomic structures have supported a monomeric fold of the mitochondrial ADP/ATP carrier [1,8,17–19]. One structural study suggested that dimerisation might be mediated via cardiolipin interactions, based upon analysis of the protein–protein interactions that form the crystal [20]. However, these interactions are weak and would provide no specificity of recognition in the presence of 50 structurally related but functionally different mitochondrial carriers present in the inner membrane. Since crystallization conditions are designed to favour protein–protein interactions, analysis of crystal packing will potentially reveal non-physiological oligomers [21]. A careful re-examination of all packing arrangements has shown that there is no conserved dimerisation interface, indicating that the carriers are structural monomers [17]. The only verified exception in the SLC25 family is the mitochondrial aspartate/glutamate carrier, which forms a structural dimer due to its unusual architecture [22]. In addition to a carrier domain, which is similar in structure to the mitochondrial ADP/ATP carrier, it has an N-terminal calcium regulatory domain and C-terminal amphipathic helix. Aspartate/glutamate carriers dimerise via their regulatory domains, as five of the eight EF-hand motifs have evolved away from their canonical calcium-binding function to form an extensive dimerisation interface. In contrast, the two carrier domains do not interact and are likely to transport as separate units [22]. The mitochondrial ATP-Mg/phosphate carrier also has a calcium regulatory domain, but it has a different architecture and is a structural monomer [23–25].

So, why did the earlier studies conclude that mitochondrial carriers are dimers? We have extensively reviewed these issues in 2010 [15], but it is worthwhile highlighting some of the key points, as the same technical mistakes are still made. First of all, it is extremely important to use a good quality starting material for studies of the oligomeric state. Mitochondrial carriers are highly dynamic membrane proteins with very few polar interactions stabilising their structures, which can cause major issues with almost every aspect of this analysis. There are many potential issues with their expression, targeting, insertion and folding, when they are produced through heterologous expression. In the Gram-negative bacterium *Escherichia coli* they end up in inclusion bodies in a misfolded and aggregated state [26], whereas in the Gram-positive bacterium *Lactococcus lactis* they are targeted to the cytoplasmic membrane [27–30], but a fraction might be misfolded [28]. Mitochondrial carriers expressed in the inner mitochondrial membrane of yeast or mammalian cells, using the endogenous synthesis, targeting, insertion and folding pathways, are the best starting point. Yeast is preferred because cultures can be scaled up easily, a requirement for studying organellar membrane proteins that are comparatively in low abundance [31–33].

## Oligomeric state of mitochondrial carriers in detergent

Purified carrier in detergent is a good starting point, as its oligomeric state can be determined away from contaminants. Detergents are required to solubilise the carrier from the lipidic membrane and to take it through several purification steps to obtain a pure sample. The choice of detergent is crucial, as harsh detergents can intercalate and unfold the protein, leading to structural instability and aggregation, an artificial state. It is also possible that detergents can separate oligomers into protomers, misrepresenting its true nature. However, if a suitably mild detergent is chosen, its atomic structure and oligomeric state are preserved, as observed for many other transporters, such as the dimeric Na^+^/H^+^ antiporter [34], Na^+^/betaine symporter BetP [35], the band 3 protein [36] and the trimeric sodium/aspartate symporter Glt_Ph_ [37]. Analysis of the oligomerization interfaces of membrane proteins shows features shared with those of soluble proteins: the interfaces tend to be large and tightly packed, involving a multitude of residues [38].

When any sizing method is used, it is extremely important to account for the contribution of the detergent/lipid micelle associated with the membrane protein. As mitochondrial carriers are relatively small, the contribution of the micelle to the total mass can be relatively large. In size exclusion chromatography, the total mass of the yeast ADP/ATP carrier can be as much as 115 kDa in dodecylmaltoside or 134 kDa in tridecylmaltoside. However, when the detergent/protein weight ratios are determined, giving 2.4 and 3.0, respectively, the carriers are clearly monomeric (33.8 and 33.5 kDa) [39,40].

In blue native gel electrophoresis, the molecular mass of mitochondrial carriers is systematically overestimated, because they run as a protein/detergent/lipid/Coomassie complex rather than a protein/Coomassie complex, as often assumed [41]. The size changes with different detergent and lipid content of the associated micelle, showing that they are key factors. By complete coincidence, the apparent mass of the protein in dodecylmaltoside comes out to be ∼66 kDa, which is roughly twice the theoretical molecular mass, but when contributions of detergent, lipid and Coomassie are taken into account, it is a monomer [41]. In the case of the mitochondrial pyruvate carrier (SLC54) in digitonin, the molecular mass was 150 kDa in blue native gel electrophoresis [42], but in reality, it is 31 kDa [43]. Thus, this method is not a reliable sizing technique for small membrane proteins [41].

Another important technical issue has been that protein assays can be adversely affected by the presence of detergents and lipids. When a protein is purified in Triton X-100, the modified Lowry assay overestimates the protein content considerably [44], which has affected the outcome of binding studies with inhibitors, resulting in inhibitor/carrier stoichiometries of 1 : 2 [45–47]. It is better to use the bicinchoninic acid assay, or better still, amino acid analysis, which provides an accurate measure of protein concentration, irrespective of the detergent and lipid content of the sample. We now know that the inhibitor/carrier stoichiometries are 1 : 1 from calorimetry measurements [48] and structural analysis [17–19]. The quantification of protein has also affected the interpretation of analytical ultracentrifugation data due to the overestimation of the protein contribution [49,50], but subsequent studies have confirmed that the carriers are monomeric using this technique [39,51].

Small-angle neutron scattering (SANS) offers the potential for determining the oligomeric state of membrane proteins in detergent solution, since contrast matching by deuterium exchange can allow the contributions from lipid and detergent to be removed from the scattering data [52]. However, bovine AAC1 in LAPAO was estimated to be 53, 56 or 61 kDa [53] and more recently 44 kDa [51], reflecting the difficulties with completely masking the contribution of lipid/detergent micelles by deuterium exchange when in large excess, leading to an overestimation of the protein mass.

The combination of size exclusion chromatography with multi-angle laser light scattering (SEC-MALLS) is a reliable method for determining the mass of membrane protein in a micelle, which can accurately account for the contributions from detergent and lipid [54]. It has been successfully applied to several mitochondrial transporters [22,43], giving the correct mass, but it is essential that they are separated fully from contaminants and free detergent micelles [54].

To analyse whether membrane proteins form a complex in detergents, one can also apply a differential tagging strategy, where tagged and untagged versions of the protein are co-expressed in the membrane and purified by affinity chromatography [55]. If they were to form a complex, the untagged proteins should co-purify with the tagged protein upon elution. For the yeast ADP/ATP carrier, all untagged carriers were found in the flow through, whereas all tagged carriers were found in the elution fraction, showing no state-dependent association even in the mildest detergents [55]. In our opinion there has never been a valid observation of a dimer of the mitochondrial ADP/ATP carrier in detergent, when all technical issues are taken into account.

## The mitochondrial ADP/ATP carrier functions as a monomer

A key question that needed to be resolved is whether the mitochondrial ADP/ATP carrier requires dimerisation for function. One observation that has been held in favour is the autosomal dominant inheritance of progressive external ophthalmoplegia, which is caused by missense mutations in the gene coding for the human ADP/ATP carrier 1, also called SLC25A4 or ANT1 [56]. However, dominant inheritance *per se* does not provide proof for dimer formation, as there are many other genetic mechanisms leading to dominant inheritance, such as (i) increased gene dosage; (ii) ectopic or temporally altered mRNA expression; (iii) increased or constitutive protein activity; (iv) altered structural proteins; (v) toxic protein alterations; (vi) new protein function; and (vii) reduced gene dosage, expression or protein activity, also called haploinsufficiency [57]. It has been estimated that the mitochondrial ADP/ATP carriers in the human body need to transport the equivalent of ∼65 kg of ADP and ATP across the inner mitochondrial membrane every day to fuel our cellular processes [58,59]. Given this enormous flux, it is likely that any failure of a fraction of the carrier population would lead to issues with mitochondrial function and energy provision, and eventually to disease, pointing to haploinsufficiency as a plausible mechanism. However, genetics alone is not sufficient to resolve these issues conclusively, as it needs to be investigated by studying negative dominance on protein function directly. In yeast, no negative dominance was observed in transport, demonstrating that the carriers function independently from each other in the mitochondrial inner membrane [60]. Furthermore, when the ADP/ATP carrier is tagged with different lengths of poly-histidine tags, the expression levels decreased with increasing tag length. The growth rates of the strains expressing these modified carriers on glycerol, a nonfermentable carbon source, decreased with decreasing expression level, demonstrating haploinsufficiency (unpublished data, Marilyn Harding).

However, the most compelling argument for the carrier being active as a monomer is that it has all of the functional features required for transport: a single central substrate-binding site and two gates with salt bridge networks that provide access to one or the other side of the membrane in an alternating way [1,8,17,18,61–64].

## The mitochondrial ADP/ATP carrier is a monomer in the membrane

The one issue that has not been resolved is the oligomeric state of the mitochondrial ADP/ATP carrier in the inner membrane. There are plenty of cases where membrane proteins or protein complexes function as monomers, but form dimers or oligomers in the membrane, such as the mitochondrial ATP synthase (dimer) [65], the glutamate transporter (trimer) [66] and aquaporin (tetramer) [67]. The mitochondrial inner membrane is one of the most densely packed of all and the mitochondrial ADP/ATP carrier is the most abundant protein therein. Even if the carrier functions as a monomer, it is still possible that it is present in some close-packing arrangement. There are very few studies that have addressed this particular possibility.

In one study, the bovine ADP/ATP carrier was reconstituted into liposomes and its diameter was determined by freeze-fracture electron microscopy, giving an average of ∼75 Å [68]. No correction for the thickness of the stain layer was carried out, which can be as much as ∼17 Å [69]. The matrix side of the bovine ADP/ATP carrier has a diameter of ∼42 Å and is the only part that protrudes significantly from the membrane [64,70]. Thus, the size of the observed particles, corrected for the contribution of the stain layer, is only compatible with a monomer. The particles are not dimeric, otherwise, they would have had a pronounced elongated appearance, which is not evident in the micrographs, nor do they form ordered clusters. In another study, high-density membranes with reconstituted yeast ADP/ATP carrier did not show any systematic dimeric arrangements, when scanned with atomic force microscopy [70]. Thus, these studies show that the carriers do not form preferred dimeric interactions in membranes after reconstitution, but they do not address what happens in the mitochondrial membrane itself.

Recently, new claims for a dimeric state of the bovine ADP/ATP carrier have emerged based on native mass spectrometry of injected mitochondrial membranes [71], although it should be noted that the method has already attracted criticism for the failure to preserve the quaternary structure and oligomeric state of mitochondrial complexes [72]. Native mass spectrometry is an established technique for the analysis of the oligomeric state and the chemical composition of tightly bound molecules to membrane proteins, purified in detergents [73–75]. These native mass spectra are complex because of the binding of lipid and detergent molecules in variable amounts, as well as ligands and contaminants. In the new approach, membrane vesicles are injected directly, giving the combined mass spectra of hundreds of different membrane proteins and membrane-associated proteins in variable relative abundance and charge. The interpretation of these spectra is extremely complex, complicated further by the association of different molecules and by post-translation modifications.

**Figure 1.**
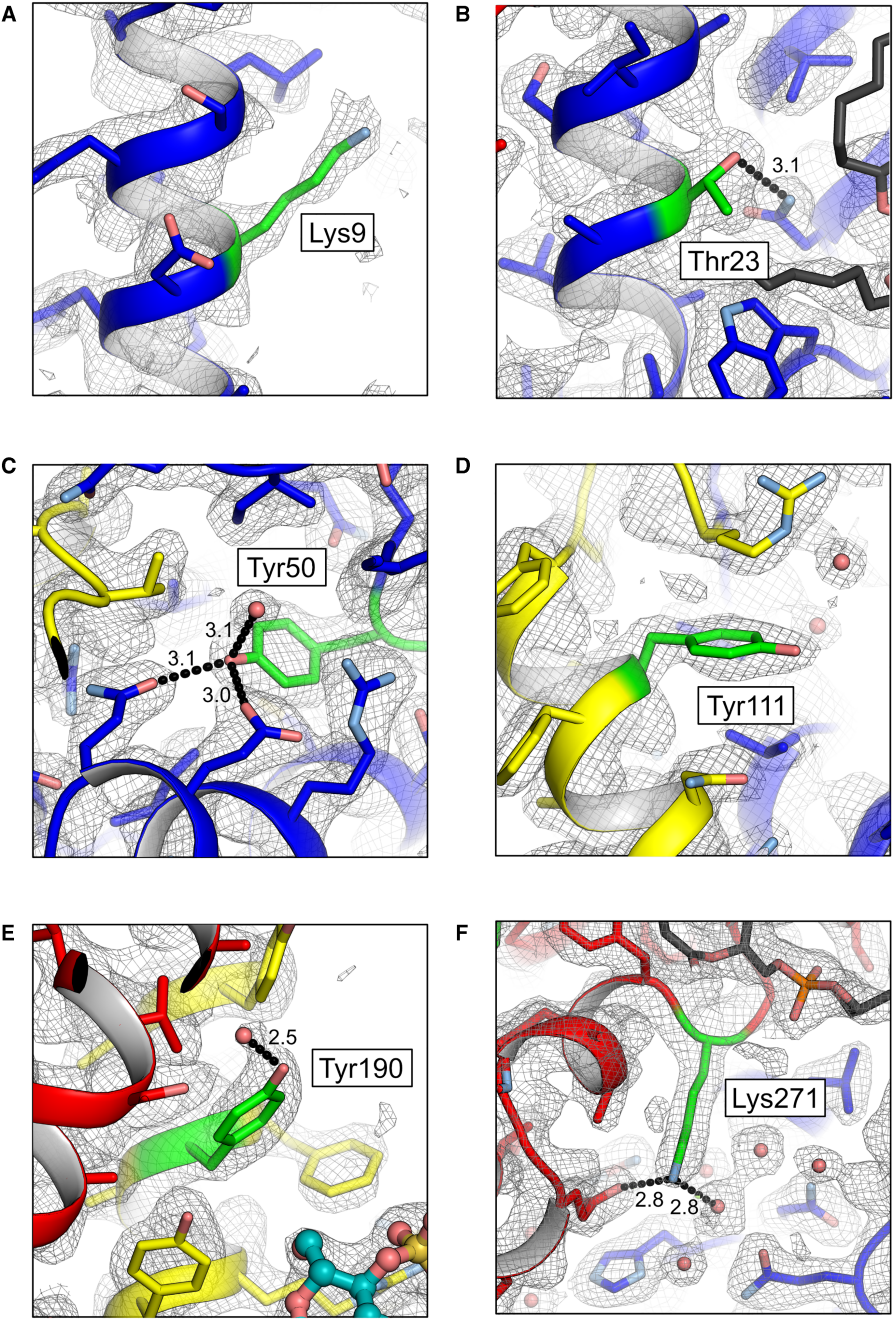
Electron density maps do not support the claimed post-translational modifications of the bovine ADP/ATP carrier. The 2mFo − DFc electron density maps around (**A**) Lys 9, (**B**) Thr23, (**C**) Tyr50, (**D**) Tyr111, (**E**) Tyr190 and (**F**) Lys271, shown as a grey mesh and contoured at the root mean square electron density (1σ), using structure factors and atomic co-ordinates from the Protein Data Bank (PDB code: 1OKC). The carrier is shown in cartoon representation with side chains as sticks, coloured by domain (domain 1, blue; domain 2, yellow; domain 3, red), whereas the residues that are claimed to be modified are shown in green. Cardiolipins are shown in dark grey and carboxyatractyloside in teal. Water molecules are shown as red spheres. Hydrogen bonds are indicated by black dashed lines, with distances indicated (Å). The side-chain of Asn73, which interacts with Thr23, has been flipped to maximise its hydrogen-bonding potential.

In the study in question, masses were assigned to both monomeric and dimeric states of the mitochondrial ADP/ATP carrier in isolated membranes from bovine heart, giving 33 195 and 66 387 Da, respectively [71], with the dimer being the most abundant form. The molecular mass of the bovine ADP/ATP carrier has been determined independently twice before. The first-ever amino acid sequence for the SLC25 family was determined by peptide sequencing of proteolytic fragments of the purified native bovine carrier. The study concluded that the mature protein lacks the N-terminal Met and has an acetylated Ser1 and a trimethylated Lys51 [9,76,77], giving a total mass of 32 921 Da. Residue numbering consequently differs from the protein encoded by the gene, but is consistent with the Protein Data Bank entries of the bovine structure (1OKC, 2C3E) [19]. The first two modifications are common in mitochondrial carriers, e.g. the uncoupling protein [10,48], and the third has been observed in other mitochondrial proteins, e.g. the c-ring of mitochondrial ATP synthase [78], for which a specific methyltransferase has been identified [79]. The second independent study carried out a total molecular mass determination of purified bovine carrier using mass spectrometry, and concluded that it was 32 925.3 ± 3.2 Da [80], which agrees with the mass of the modified protein determined from the first study. Thus, there is a clear discrepancy between the mass observed in native mass spectrometry [71] and the one determined by protein sequencing [9] and mass spectrometry [80], which is not accounted for by experimental error. In a subsequent study, this discrepancy was attributed to a large number of post-translational modifications [81]. In addition to the ones mentioned above, they observed *O*-acetylation of another four residues, Thr23, Tyr50, Tyr111 and Tyr190, *N*-succinylation of Lys22 and Lys271, and *N*-trimethylation of Lys9. They note that there might be other modifications, but only the ones with high confidence are reported. This extensively modified carrier must be the predominant species in the membrane, as no other was discernable in the native mass spectrometry data. Since high-resolution structures of the bovine ADP/ATP carrier have been solved, we checked whether there is any evidence for the additional post-translational modifications in the electron density maps [19,20]. In any crystal structure, regions of a protein can be disordered and hence show weak or no electron density, which cannot be reliably modelled. To avoid these issues, we have focussed on the structure solved at the highest resolution (2.2 Å, PDB code: 1OKC), and on residues that lie in well-ordered regions, with low B-factors, where the electron density map shows clear density for backbone carbonyl oxygens and water molecules. A careful inspection of these sites shows that there are no additional unexplained electron densities near the side chains of these residues, and hence no evidence for their post-translational modification (Figure 1). To exclude the remote possibility that the bulk-solvent model used in crystallographic refinement and map calculation might have flattened the electron density near the side chains, we have calculated a polder OMIT map for each residue [82]. These maps confirm that there is no crystallographic support for the proposed post-translational modifications. Thus, the data from three different techniques are consistent with each other with respect to the mass and level of modification [9,76,80], but not with those observed by native mass spectrometry [71,81].

Next, we inspected the sites of the proposed modifications in the context of the structure and mechanism of the ADP/ATP carrier. In the structure, residues Thr23, Tyr50 and Lys271 are involved in key interactions that stabilise the domain structures, and their modification would lead to disruption and destabilisation of the carrier (Figure 1). Tyr50, which is bonded to two other residues, is in a structural environment that could not possibly accommodate an acetylation modification. Thr23 is inaccessible for modification in the cytoplasmic-state, where the side-chain is packed between a neighbouring transmembrane α-helix and the lipid bilayer, and in the matrix-state, where it is buried in the bilayer [18,19]. Therefore, Thr23 and Tyr50 could only become modified when the protein is unfolded.

**Figure 2.**
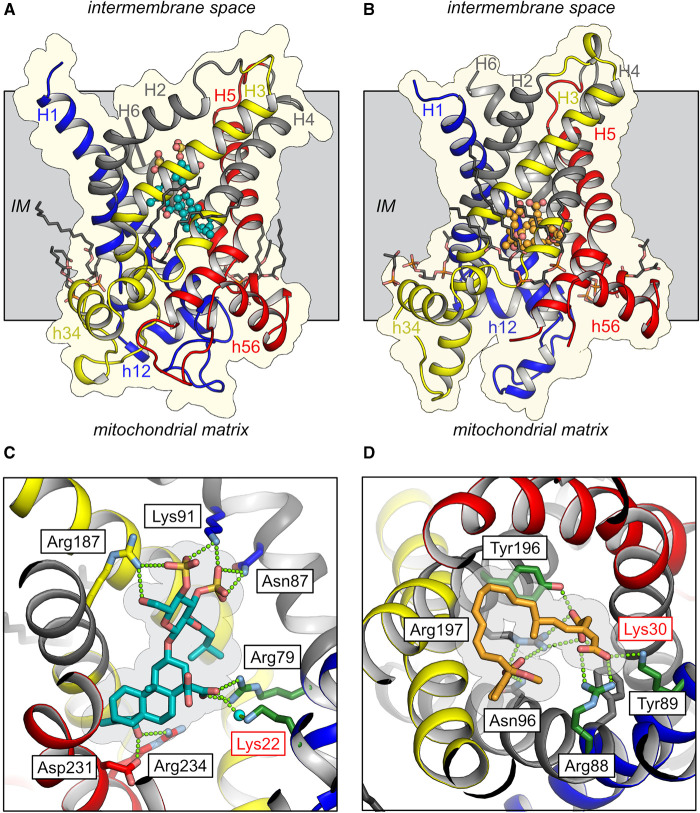
Structures of mitochondrial ADP/ATP carriers inhibited by specific inhibitors. (**A**) Lateral view of the structure of the bovine ADP/ATP carrier (PDB code: 1OKC) trapped in the cytoplasmic-state by the inhibitor carboxyatractyloside (CATR), which was refined with the correct stereo-isoform of CATR [107]. (**B**) Lateral view of the structure of the ADP/ATP carrier of *Thermothelomyces thermophila* (PDB code: 6GCI) trapped in the matrix-state by the inhibitor bongkrekic acid (BKA). The carriers are shown in cartoon representation coloured by domain (blue, domain 1; yellow, domain 2; red, domain 3) and with the gate elements on the even-numbered transmembrane α-helices in grey. Details of protein-inhibitor interactions are shown in (**C**) for CATR and (**D**) for BKA. The lysine residue that is claimed to be succinylated is indicated by a red label. The inhibitors are shown in teal for CATR and in orange for BKA, whereas cardiolipin molecules are shown in grey. Polar interactions between protein and inhibitors are indicated by green dotted lines. IM mitochondrial inner membrane.

**Figure 3.**
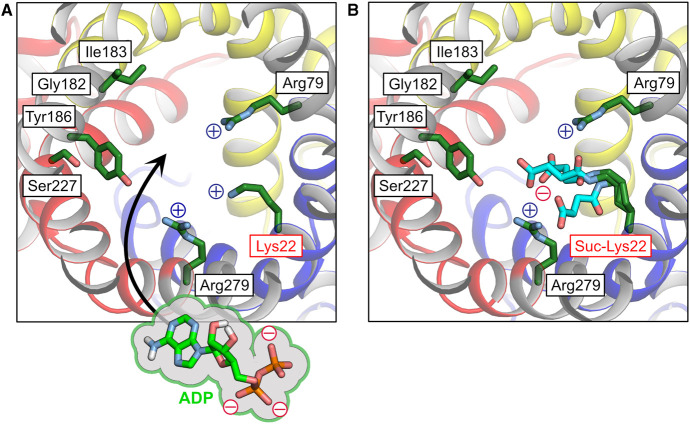
Succinylation of Lys22 would interfere with substrate binding. (**A**) Residues of the proposed substrate-binding site, shown in green, in the cytoplasmic state of the bovine ADP/ATP carrier (PDB:1OKC) [19]. The three positively charged residues (Lys22, Arg79, Arg279) are predicted to form salt bridge interactions with the negatively charged phosphate groups of the substrates ADP and ATP. Gly182, Ile183, Tyr186 and Ser227 form the adenine binding pocket. ADP is shown in stick and surface representation at the van der Waals radii of the atoms, for comparison. (**B**) The proposed post-translational succinylation of Lys22 (Suc-Lys22), here represented by the three possible conformers, would sterically hinder substrate binding and significantly alter the electrostatic potential of the binding site by removing a positive charge and by introducing a negative charge. Moreover, the succinyl modification could form an ionic interaction with either Arg79 or Arg279, removing two of the three positive charges from the site, compromising the binding of the negatively charged ADP^3−^ or ATP^4−^.

To justify the assignment of the 33 195 Da mass to the ADP/ATP carrier, control experiments were performed in which two specific inhibitors were added to the membrane prior to analysis by native mass spectrometry [81]. The experimental conditions are not described, and a concentration dependency of binding was not determined, and thus it is impossible to verify whether the observed low-level binding is specific or non-specific. However, we can confirm that *N*-succinylation of Lys22 would interfere with the binding of these inhibitors, as the modification would be located in their binding pockets (Figure 2). The amine group of Lys22 binds via an ordered water to the carboxyl group of carboxyatractyloside in the cytoplasmic state of the bovine carrier [17,19] (Figure 2C). In the matrix state structure of the ADP/ATP carrier of *Thermothelomyces thermophila*, Lys30, the equivalent residue of Lys22, binds directly to a carboxyl group of bongkrekic acid [18] (Figure 2D). So, in both cases, the proposed modification would abolish these interactions. The positively charged Lys22 is also a key residue in the binding of the negatively charged substrates ADP and ATP (Figure 3) [8,61–64,83–85]. *N*-succinylation of Lys22 would (i) introduce a large group in the binding pocket of the substrates and inhibitors, (ii) abolish a positive charge providing key ionic interactions with their negatively charged functional groups and (iii) introduce a negatively charged modification where the negatively charged compounds are supposed to bind, creating electrostatic repulsion. It is also possible that the negatively charged succinyl modification could form ionic interactions with either Arg79 or Arg279, removing two of the three positive charges from the site, which are crucial for the binding of the substrates. Thus, the highly modified carrier could not be in a folded and functional state, capable of binding inhibitors or substrates.

**Figure 4.**
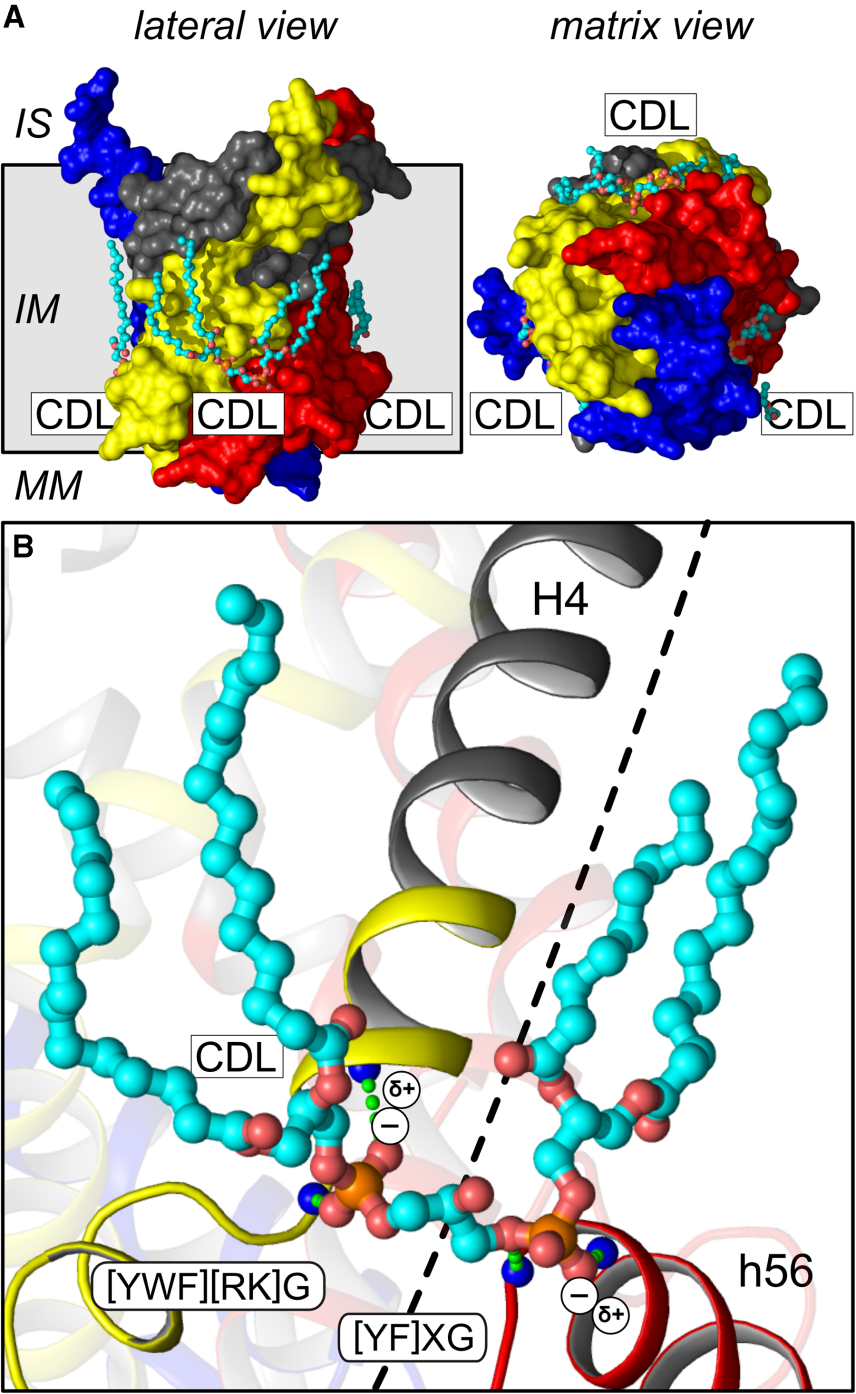
Three cardiolipin molecules are tightly bound to the mitochondrial ADP/ATP carrier. (**A**) Side and matrix view of bovine adenine nucleotide translocase (2CE3), showing three tightly bound cardiolipin molecules. Domains 1, 2 and 3 are shown in blue, yellow and red, respectively, and the gate elements in grey. The cardiolipin molecules are shown in cyan and their acyl chains have only been partially modelled. (**B**) The negatively charged cardiolipin molecules are tightly bound by electrostatic interactions with the helix dipoles, which have a formal positive charge (δ+), and by hydrogen bonds between the phosphate moieties and amide groups (blue spheres) of the even-numbered transmembrane helices, here H4, and matrix helices, here h56. The cardiolipin binding sites are highly conserved and the consensus sequence for the even-numbered helices (left) and matrix helices (right) is shown. The dashed line represents the inter-domain interface. IS, intermembrane space; IM inner membrane; MM mitochondrial matrix; CDL cardiolipin molecule.

A large number of independent studies have reported the importance of three tightly bound cardiolipin molecules in the structure and transport mechanism of mitochondrial carriers, including phosphorous NMR [86], crystallographic analyses [17–19], lipid analysis [39,40,48,87], disease models [88–90], thermostability measurements [91], transport assays [83,92,93] and coarse grain and molecular dynamics simulations [94–96]. The measured mass of 33 195 Da, assigned to the bovine ADP/ATP carrier by native mass spectrometry, does not account for the binding of three cardiolipin molecules, otherwise, it would have been 37 271 Da. Moreover, the spectrum does not show the typical fine-splitting of the native mass peak due to bound cardiolipin or other lipid molecules having acyl chains of differing length and saturation. To explain the missing cardiolipin molecules, it was incorrectly asserted that the cardiolipins observed in the crystal structures were present because cardiolipin was added during purification and crystallisation trials [81]. Actually, in most cases, the carrier was isolated directly from the mitochondrial inner membrane in detergent solutions without cardiolipin addition [17,19,20]. Cardiolipin molecules are so tightly bound that extensive washing with detergents does not remove them [39,48]. Cardiolipin binding occurs at three specific sites in the mitochondrial ADP/ATP carrier, which have special properties (Figure 4A). The two negatively charged phosphate moieties of cardiolipin are bound between the N-terminus of the even-numbered helix of one domain and the N-terminus of the matrix helix of the proximal domain. They are bonded by hydrogen bonds [19,20] and by electrostatic interactions with the positive helix dipole [17,18,64], explaining their high-affinity binding (Figure 4B). For these reasons, the loop to helix transitions have highly conserved [YF]xG and [YWF][RK]G motifs, where the glycine residues function as helix breakers [18,64,97]. The bound cardiolipin molecules are important for the stability and function of the carrier, as they link two adjacent domains together when the carrier cycles between the matrix and cytoplasmic state.

Since the carrier is relatively small and embedded in a lipid bilayer, and has short loops, most of the protein is not accessible for enzyme-mediated post-translational modifications. However, reactive metabolites, such as acetyl-CoA and succinyl-CoA, can potentially directly react with amino acid side chains [98], which could occur more readily in the mitochondrial matrix, which has an alkaline pH and relatively high concentrations of these compounds [99–101]. Non-enzymatic acetylation and succinylation of lysines in mitochondrial proteins has been reported, but they occur with low stoichiometry [102,103]. The acetylation of threonine and tyrosine residues has been described, but also appears to be rare [104,105]. If real, the modified residues would be inaccessible to enzymes and there is no known non-enzymatic mechanism for removal of the modifications, so this cannot be regulatory.

So, how could the highly modified carrier become the dominant form in these studies, whereas it is not observed at all in other analyses? Significantly, prior to mass spectrometry, the membranes were sonicated in 0.5 M of ammonium acetate buffer to remove bound ions and to facilitate extraction of proteins from the membrane [106]. The sonication procedures and the high voltages used for electrospray ionisation will have deposited a lot of energy into the sample, which may explain the unusually high level of modifications. Consistent with this, the experimental procedures have also led to the disruption of the mitochondrial ATP synthase and complex III dimers, to the removal of a large number of subunits from complex I and III, and to the complete removal of lipids, normally associated with membrane proteins [72]. The disrupted interaction surfaces of the mitochondrial complexes are many orders of magnitude larger than those of any hypothetical carrier dimer, which would only contain a few amino acids. The protein, assigned to the ADP/ATP carrier by native mass spectrometry, seems to lack all of the known properties of the native one in the inner membrane of mitochondria, i.e. molecular mass, level of modification and cardiolipin binding. If we assume the assignment is correct, these treatments have led to structural destabilisation, hyper-modification and delipidation of the mitochondrial ADP/ATP carrier, which could lead to non-specific aggregation. Under these conditions, any assessment of the oligomeric state becomes pointless, as the native state and function of these highly labile proteins have not been preserved by the method of analysis.

## Simulations addressing the oligomeric state of the mitochondrial ADP/ATP carrier

Coarse grain simulations have been used to study the behaviour of an ensemble of mitochondrial ADP/ATP carriers in simulated membranes [71,95]. For these analyses, the carboxyatractyloside-inhibited state of the ADP/ATP carrier was used, after the removal of the inhibitor. The inhibitor locks the carrier in an abortive state, which is not part of the transport cycle [6]. Comparisons to the bongkrekic acid-inhibited state have shown that there are some structural perturbations induced by inhibitor binding consistent with the state being abortive [18]. Thus, the question is whether it was a good starting point for this type of analysis. In reality, the carriers cycle continuously between a cytoplasmic and matrix conformation, each of them being in a different state, and this currently cannot be modelled accurately. Nevertheless, in these simulations, the carriers form dimers, trimers, tetramers and higher-order aggregates. Notably, they form predominantly if cardiolipin was present in the inner leaflet of the membrane [71]. However, and most importantly, the carriers do not associate in a consistent way and do not have a conserved dimerisation interface. Associations occur on all sides of the carriers without specificity, roughly following the three-fold pseudo-symmetrical shape of the carrier. Rather than forming specific dimers, the carriers are arranged in closely-packed clusters without conserved interactions.

## New insight from structural analysis

In the functional mitochondrion, mitochondrial carriers cycle back and forth between the cytoplasmic and matrix state, both of which have been characterised structurally for the ADP/ATP carrier (Figure 2A,B) [17–19]. The conformational changes involve six elements, which move concomitantly [8,18]. There are three core elements, which consist of the odd-numbered helices, the matrix helices and one-third of the even-numbered helices (primary colours, Figure 2A,B) plus three gate elements, which are the C-terminal ends of the even-numbered helices in each domain (grey colours, Figure 2A,B) [8,18]. During the transition from the cytoplasmic to the matrix state, the core elements of each domain rock outwards, opening the matrix side of the carrier, whereas simultaneously the gate elements rotate inwards, closing the cytoplasmic side of the carrier (Figure 2A,B). The transition between the matrix and cytoplasmic state involves the movement of the same elements, but in reverse [8,18]. The conformational changes lead to profound changes in shape that are totally incompatible with a stable dimerisation interface (Figure 2A,B) [63]. Small movements in the substrate-binding site [61–63] translate to large movements on the periphery of the carrier, some as much as 13 Å [18]. The inner membrane of a fully functional mitochondrion contains 50 different mitochondrial carriers, each in a different conformational state in the transport cycle at any one time. The carriers cycle between these states approximately a thousand times per second. With this in mind, and all the other arguments presented in this review, it is simply not possible that they form stable structural dimers during transport of their substrates.

## Perspectives

- **Importance of the field:** The mitochondrial ADP/ATP carrier performs one of the most important transport steps in the human body, transporting the spent fuel ADP into the mitochondrion and the synthesised fuel ATP out to energise processes in the cell.
- **Current thinking:** Originally it was thought that they were dimers, but all of the sequence, structural, biophysical and functional data now show that the mitochondrial ADP/ATP carrier exists and functions as a monomer.
- **Future directions:** Many structural aspects of the transport mechanism still need to be resolved to determine how mitochondrial ADP/ATP carriers achieve high transport rates of adenine nucleotides in the densely packed inner membrane of mitochondria.

## Open Access

Open access for this article was enabled by the participation of University of Cambridge in an all-inclusive *Read & Publish* pilot with Portland Press and the Biochemical Society under a transformative agreement with JISC.

## Abbreviations

SANS: small-angle neutron scattering
SEC-MALLS: size exclusion chromatography with multi-angle laser light scattering

## References

[1] Ruprecht J.J. and Kunji E.R.S. (2019) The SLC25 mitochondrial carrier family: structure and mechanism. Trends Biochem. Sci. 45, 244–258 10.1016/j.tibs.2019.11.001 31787485 PMC7611774

[2] Kunji E.R.S. (2012) Structural and mechanistic aspects of mitochondrial transport proteins In Comprehensive Biophysics (Ferguson S., ed.), pp. 174–205, Elsevier

[3] Kunji E.R.S., King M.S., Ruprecht J.J. and Thangaratnarajah C. (2020) The SLC25 carrier family: important transport proteins in mitochondrial physiology and pathology. Physiology, in press 10.1152/physiol.00009.2020 PMC7611780 32783608

[4] Palmieri F., Scarcia P. and Monne M. (2020) Diseases caused by mutations in mitochondrial carrier genes SLC25: a review. Biomolecules 10, 655 10.3390/biom10040655 PMC7226361 32340404

[5] Palmieri F. and Monne M. (2016) Discoveries, metabolic roles and diseases of mitochondrial carriers: a review. Biochim. Biophys. Acta 1863, 2362–2378 10.1016/j.bbamcr.2016.03.007 26968366

[6] Klingenberg M. (2008) The ADP and ATP transport in mitochondria and its carrier. Biochim. Biophys. Acta 1778, 1978–2021 10.1016/j.bbamem.2008.04.011 18510943

[7] Pebay-Peyroula E. and Brandolin G. (2004) Nucleotide exchange in mitochondria: insight at a molecular level. Curr. Opin. Struct. Biol. 14, 420–425 10.1016/j.sbi.2004.06.009 15313235

[8] Ruprecht J.J. and Kunji E.R.S. (2019) Structural changes in the transport cycle of the mitochondrial ADP/ATP carrier. Curr. Opin. Struct. Biol. 57, 135–144 10.1016/j.sbi.2019.03.029 31039524 PMC6700394

[9] Aquila H., Misra D., Eulitz M. and Klingenberg M. (1982) Complete amino acid sequence of the ADP/ATP carrier from beef heart mitochondria. Hoppe Seylers Z Physiol. Chem. 363, 345–349 10.1515/bchm2.1982.363.1.345 7076130

[10] Aquila H., Link T.A. and Klingenberg M. (1985) The uncoupling protein from brown fat mitochondria is related to the mitochondrial ADP/ATP carrier. EMBO J. 4, 2369–2376 10.1002/j.1460-2075.1985.tb03941.x 3000775 PMC554512

[11] Runswick M.J., Powell S.J., Nyren P. and Walker J.E. (1987) Sequence of the bovine mitochondrial phosphate carrier protein: structural relationship to ADP/ATP translocase and the brown fat mitochondria uncoupling protein. EMBO J. 6, 1367–1373 10.1002/j.1460-2075.1987.tb02377.x 3038521 PMC553942

[12] Saraste M. and Walker J.E. (1982) Internal sequence repeats and the path of polypeptide in mitochondrial ADP/ATP translocase. FEBS Lett. 144, 250–254 10.1016/0014-5793(82)80648-0 6288471

[13] Walker J.E. (1992) The mitochondrial transporter family. Curr. Opin. Struct. Biol. 2, 519–526 10.1016/0959-440X(92)90081-H

[14] Klingenberg M. (1981) Membrane protein oligomeric structure and transport function. Nature 290, 449–454 10.1038/290449a0 6261141

[15] Kunji E.R.S. and Crichton P.G. (2010) Mitochondrial carriers function as monomers. Biochim. Biophys. Acta 1797, 817–831 10.1016/j.bbabio.2010.03.023 20362544

[16] Kunji E.R.S. and Harding M. (2003) Projection structure of the atractyloside-inhibited mitochondrial ADP/ATP carrier of *Saccharomyces cerevisiae*. J. Biol. Chem. 278, 36985–36988 10.1074/jbc.C300304200 12893834

[17] Ruprecht J.J., Hellawell A.M., Harding M., Crichton P.G., Mccoy A.J. and Kunji E.R.S. (2014) Structures of yeast mitochondrial ADP/ATP carriers support a domain-based alternating-access transport mechanism. Proc. Natl. Acad. Sci. U.S.A. 111, E426–E434 10.1073/pnas.1320692111 24474793 PMC3910652

[18] Ruprecht J.J., King M.S., Zogg T., Aleksandrova A.A., Pardon E., Crichton P.G. et al. (2019) The molecular mechanism of transport by the mitochondrial ADP/ATP carrier. Cell 176, 435–447 10.1016/j.cell.2018.11.025 30611538 PMC6349463

[19] Pebay-Peyroula E., Dahout-Gonzalez C., Kahn R., Trezeguet V., Lauquin G.J. and Brandolin G. (2003) Structure of mitochondrial ADP/ATP carrier in complex with carboxyatractyloside. Nature 426, 39–44 10.1038/nature02056 14603310

[20] Nury H., Dahout-Gonzalez C., Trezeguet V., Lauquin G., Brandolin G. and Pebay-Peyroula E. (2005) Structural basis for lipid-mediated interactions between mitochondrial ADP/ATP carrier monomers. FEBS Lett. 579, 6031–6036 10.1016/j.febslet.2005.09.061 16226253

[21] Dafforn T.R. (2007) So how do you know you have a macromolecular complex? Acta Crystallogr. D Biol. Crystallogr. 63, 17–25 10.1107/S0907444906047044 17164522 PMC2483502

[22] Thangaratnarajah C., Ruprecht J.J. and Kunji E.R.S. (2014) Calcium-induced conformational changes of the regulatory domain of human mitochondrial aspartate/glutamate carriers. Nat. Commun. 5, 5491 10.1038/ncomms6491 25410934 PMC4250520

[23] Harborne S.P., Ruprecht J.J. and Kunji E.R.S. (2015) Calcium-induced conformational changes in the regulatory domain of the human mitochondrial ATP-Mg/Pi carrier. Biochim. Biophys. Acta 1847, 1245–1253 10.1016/j.bbabio.2015.07.002 26164100 PMC4562336

[24] Harborne S.P., King M.S., Crichton P.G. and Kunji E.R.S. (2017) Calcium regulation of the human mitochondrial ATP-Mg/Pi carrier SLC25A24 uses a locking pin mechanism. Sci. Rep. 7, 45383 10.1038/srep45383 28350015 PMC5369052

[25] Harborne S.P.D. and Kunji E.R.S. (2018) Calcium-regulated mitochondrial ATP-Mg/Pi carriers evolved from a fusion of an EF-hand regulatory domain with a mitochondrial ADP/ATP carrier-like domain. IUBMB Life 70, 1222–1232 10.1002/iub.1931 30281880 PMC6283063

[26] Fiermonte G., Walker J.E. and Palmieri F. (1993) Abundant bacterial expression and reconstitution of an intrinsic membrane-transport protein from bovine mitochondria. Biochem. J. 294, 293–299 10.1042/bj2940293 8363582 PMC1134597

[27] Kunji E.R.S., Chan K.W., Slotboom D.J., Floyd S., O'Connor R. and Monné M. (2005) Eukaryotic membrane protein overproduction in *lactococcus lactis*. Curr. Opin. Biotechnol. 16, 546–551 10.1016/j.copbio.2005.08.006 16143505

[28] Monné M., Chan K.W., Slotboom D.J. and Kunji E.R.S. (2005) Functional expression of eukaryotic membrane proteins in *Lactococcus lactis*. Protein Sci. 14, 3048–3056 10.1110/ps.051689905 16260761 PMC2253241

[29] King M.S., Boes C. and Kunji E.R.S. (2015) Membrane protein expression in *Lactococcus lactis*. Methods Enzymol. 556, 77–97 10.1016/bs.mie.2014.12.009 25857778

[30] Seigneurin-Berny D., King M.S., Sautron E., Moyet L., Catty P., Andre F. et al. (2016) Membrane protein production in *Lactococcus lactis* for functional studies. Methods Mol. Biol. 1432, 79–101 10.1007/978-1-4939-3637-3_6 27485331

[31] King M.S. and Kunji E.R.S. (2020) Expression and purification of membrane proteins in *Saccharomyces cerevisiae*. Methods Mol. Biol. 2127, 47–61 10.1007/978-1-0716-0373-4_4 32112314

[32] Hashimoto M., Shinohara Y., Majima E., Hatanaka T., Yamazaki N. and Terada H. (1999) Expression of the bovine heart mitochondrial ADP/ATP carrier in yeast mitochondria: significantly enhanced expression by replacement of the N-terminal region of the bovine carrier by the corresponding regions of the yeast carriers. Biochim. Biophys. Acta 1409, 113–124 10.1016/S0005-2728(98)00155-8 9878703

[33] Fiore C., Trezeguet V., Roux P., Le Saux A., Noel F., Schwimmer C. et al. (2000) Purification of histidine-tagged mitochondrial ADP/ATP carrier: influence of the conformational states of the C-terminal region. Protein Expr. Purif. 19, 57–65 10.1006/prep.2000.1213 10833391

[34] Hunte C., Screpanti E., Venturi M., Rimon A., Padan E. and Michel H. (2005) Structure of a Na^+^/H^+^ antiporter and insights into mechanism of action and regulation by pH. Nature 435, 1197–1202 10.1038/nature03692 15988517

[35] Ressl S., Terwisscha van Scheltinga A.C., Vonrhein C., Ott V. and Ziegler C. (2009) Molecular basis of transport and regulation in the Na(+)/betaine symporter BetP. Nature 458, 47–52 10.1038/nature07819 19262666

[36] Arakawa T., Kobayashi-Yurugi T., Alguel Y., Iwanari H., Hatae H., Iwata M. et al. (2015) Crystal structure of the anion exchanger domain of human erythrocyte band 3. Science 350, 680–684 10.1126/science.aaa4335 26542571

[37] Yernool D., Boudker O., Jin Y. and Gouaux E. (2004) Structure of a glutamate transporter homologue from *pyrococcus horikoshii*. Nature 431, 811–818 10.1038/nature03018 15483603

[38] Duarte J.M., Biyani N., Baskaran K. and Capitani G. (2013) An analysis of oligomerization interfaces in transmembrane proteins. BMC Struct. Biol. 13, 21 10.1186/1472-6807-13-21 24134166 PMC4015793

[39] Bamber L., Harding M., Butler P.J.G. and Kunji E.R.S. (2006) Yeast mitochondrial ADP/ATP carriers are monomeric in detergents. Proc. Natl. Acad. Sci. U.S.A. 103, 16224–16229 10.1073/pnas.0607640103 17056710 PMC1618811

[40] Kunji E.R.S., Harding M., Butler P.J.G. and Akamine P. (2008) Determination of the molecular mass and dimensions of membrane proteins by size exclusion chromatography. Methods 46, 62–72 10.1016/j.ymeth.2008.10.020 18952172

[41] Crichton P.G., Harding M., Ruprecht J.J., Lee Y. and Kunji E.R.S. (2013) Lipid, detergent, and Coomassie Blue G-250 affect the migration of small membrane proteins in blue native gels; mitochondrial carriers migrate as monomers not dimers. J. Biol. Chem. 288, 22163–22173 10.1074/jbc.M113.484329 23744064 PMC3724668

[42] Bricker D.K., Taylor E.B., Schell J.C., Orsak T., Boutron A., Chen Y.C. et al. (2012) A mitochondrial pyruvate carrier required for pyruvate uptake in yeast, Drosophila, and humans. Science 337, 96–100 10.1126/science.1218099 22628558 PMC3690818

[43] Tavoulari S., Thangaratnarajah C., Mavridou V., Harbour M.E., Martinou J.C. and Kunji E.R.S. (2019) The yeast mitochondrial pyruvate carrier is a hetero-dimer in its functional state. EMBO J. 38, e100785 10.15252/embj.2018100785 30979775 PMC6517818

[44] Rodriguez-Vico F., Martinez-Cayuela M., Zafra M.F., Garcia-Peregrin E. and Ramirez H. (1991) A procedure for the simultaneous determination of lipid and protein in biomembranes and other biological samples. Lipids 26, 77–80 10.1007/BF02544029 2051888

[45] Riccio P., Aquila H. and Klingenberg M. (1975) Purification of the carboxy-atractylate binding protein from mitochondria. FEBS Lett. 56, 133–138 10.1016/0014-5793(75)80127-X 1171780

[46] Lin C.S. and Klingenberg M. (1980) Isolation of the uncoupling protein from brown adipose tissue mitochondria. FEBS Lett. 113, 299–303 10.1016/0014-5793(80)80613-2 7389900

[47] Aquila H., Eiermann W., Babel W. and Klingenberg M. (1978) Isolation of the ADP/ATP translocator from beef heart mitochondria as the bongkrekate-protein complex. Eur. J. Biochem. 85, 549–560 10.1111/j.1432-1033.1978.tb12270.x 648534

[48] Lee Y., Willers C., Kunji E.R.S. and Crichton P.G. (2015) Uncoupling protein 1 binds one nucleotide per monomer and is stabilized by tightly bound cardiolipin. Proc. Natl. Acad. Sci. U.S.A. 112, 6973–6978 10.1073/pnas.1503833112 26038550 PMC4460439

[49] Lin C.S., Hackenberg H. and Klingenberg E.M. (1980) The uncoupling protein from brown adipose tissue mitochondria is a dimer. A hydrodynamic study. FEBS Lett. 113, 304–306 10.1016/0014-5793(80)80614-4 7389901

[50] Hackenberg H. and Klingenberg M. (1980) Molecular weight and hydrodynamic parameters of the adenosine 5′-diphosphate–adenosine 5′-triphosphate carrier in Triton X-100. Biochemistry 19, 548–555 10.1021/bi00544a024 6243949

[51] Nury H., Manon F., Arnou B., le Maire M., Pebay-Peyroula E. and Ebel C. (2008) Mitochondrial bovine ADP/ATP carrier in detergent is predominantly monomeric, but also forms multimeric species. Biochemistry 43, 12319–12331 10.1021/bi801053m 18980386

[52] Mahieu E. and Gabel F. (2018) Biological small-angle neutron scattering: recent results and development. Acta Crystallogr. D Struct. Biol. 74, 715–726 10.1107/S2059798318005016 30082507

[53] Block M.R., Zaccai G., Lauquin G.J. and Vignais P.V. (1982) Small angle neutron scattering of the mitochondrial ADP/ATP carrier protein in detergent. Biochem. Biophys. Res. Commun. 109, 471–477 10.1016/0006-291X(82)91745-4 6295398

[54] Slotboom D.J., Duurkens R.H., Olieman K. and Erkens G.B. (2008) Static light scattering to characterize membrane proteins in detergent solution. Methods 46, 73–82 10.1016/j.ymeth.2008.06.012 18625320

[55] Bamber L., Slotboom D.J. and Kunji E.R.S. (2007) Yeast mitochondrial ADP/ATP carriers are monomeric in detergents as demonstrated by differential affinity purification. J. Mol. Biol. 371, 388–395 10.1016/j.jmb.2007.05.072 17572439

[56] Kaukonen J., Juselius J.K., Tiranti V., Kyttala A., Zeviani M., Comi G.P. et al. (2000) Role of adenine nucleotide translocator 1 in mtDNA maintenance. Science 289, 782–785 10.1126/science.289.5480.782 10926541

[57] Wilkie A.O. (1994) The molecular basis of genetic dominance. J. Med. Genet. 31, 89–98 10.1136/jmg.31.2.89 8182727 PMC1049666

[58] Garrett R. and Grisham C.M. (2010) Biochemistry, 4th edn, International edn, Brooks/Cole; Cengage Learning, Australia; U.K

[59] Buono M.J. and Kolkhorst F.W. (2001) Estimating ATP resynthesis during a marathon run: a method to introduce metabolism. Adv. Physiol. Educ. 25, 70–71 10.1152/advances.2001.25.2.70

[60] Bamber L., Harding M., Monné M., Slotboom D.J. and Kunji E.R.S. (2007) The yeast mitochondrial ADP/ATP carrier functions as a monomer in mitochondrial membranes. Proc. Natl. Acad. Sci. U.S.A. 104, 10830–10834 10.1073/pnas.0703969104 17566106 PMC1891095

[61] Kunji E.R.S. and Robinson A.J. (2006) The conserved substrate binding site of mitochondrial carriers. Biochim. Biophys. Acta 1757, 1237–1248 10.1016/j.bbabio.2006.03.021 16759636

[62] Robinson A.J. and Kunji E.R.S. (2006) Mitochondrial carriers in the cytoplasmic state have a common substrate binding site. Proc. Natl. Acad. Sci. U.S.A. 103, 2617–2622 10.1073/pnas.0509994103 16469842 PMC1413793

[63] Robinson A.J., Overy C. and Kunji E.R.S. (2008) The mechanism of transport by mitochondrial carriers based on analysis of symmetry. Proc. Natl. Acad. Sci. U.S.A. 105, 17766–17771 10.1073/pnas.0809580105 19001266 PMC2582046

[64] Kunji E.R.S., Aleksandrova A., King M.S., Majd H., Ashton V.L., Cerson E. et al. (2016) The transport mechanism of the mitochondrial ADP/ATP carrier. Biochim. Biophys. Acta 1863, 2379–2393 10.1016/j.bbamcr.2016.03.015 27001633

[65] Davies K.M., Anselmi C., Wittig I., Faraldo-Gomez J.D. and Kühlbrandt W. (2012) Structure of the yeast F1Fo-ATP synthase dimer and its role in shaping the mitochondrial cristae. Proc. Natl. Acad. Sci. U.S.A. 109, 13602–13607 10.1073/pnas.1204593109 22864911 PMC3427116

[66] Yernool D., Boudker O., Folta-Stogniew E. and Gouaux E. (2003) Trimeric subunit stoichiometry of the glutamate transporters from *Bacillus caldotenax* and *Bacillus stearothermophilus*. Biochemistry 42, 12981–12988 10.1021/bi030161q 14596613

[67] Walz T., Hirai T., Murata K., Heymann J.B., Mitsuoka K., Fujiyoshi Y. et al. (1997) The three-dimensional structure of aquaporin-1. Nature 387, 624–627 10.1038/42512 9177353

[68] Brandolin G., Doussiere J., Gulik A., Gulik-Krzywicki T., Lauquin G.J. and Vignais P.V. (1980) Kinetic, binding and ultrastructural properties of the beef heart adenine nucleotide carrier protein after incorporation into phospholipid vesicles. Biochim. Biophys. Acta 592, 592–614 10.1016/0005-2728(80)90103-6 6251872

[69] Eskandari S., Wright E.M., Kreman M., Starace D.M. and Zampighi G.A. (1998) Structural analysis of cloned plasma membrane proteins by freeze-fracture electron microscopy. Proc. Natl. Acad. Sci. U.S.A. 95, 11235–11240 10.1073/pnas.95.19.11235 9736719 PMC21625

[70] Kedrov A., Hellawell A.M., Klosin A., Broadhurst R.B., Kunji E.R.S. and Muller D.J. (2010) Probing the interactions of carboxy-atractyloside and atractyloside with the yeast mitochondrial ADP/ATP carrier. Structure 18, 39–46 10.1016/j.str.2009.11.009 20152151

[71] Chorev D.S., Baker L.A., Wu D., Beilsten-Edmands V., Rouse S.L., Zeev-Ben-Mordehai T. et al. (2018) Protein assemblies ejected directly from native membranes yield complexes for mass spectrometry. Science 362, 829–834 10.1126/science.aau0976 30442809 PMC6522346

[72] Hirst J., Kunji E.R.S. and Walker J.E. (2019) Comment on “Protein assemblies ejected directly from native membranes yield complexes for mass spectrometry”. Science 366, eaaw9830 10.1126/science.aaw9830 31699909

[73] Mehmood S., Marcoux J., Gault J., Quigley A., Michaelis S., Young S.G. et al. (2016) Mass spectrometry captures off-target drug binding and provides mechanistic insights into the human metalloprotease ZMPSTE24. Nat. Chem. 8, 1152–1158 10.1038/nchem.2591 27874871 PMC5123592

[74] Liko I., Allison T.M., Hopper J.T. and Robinson C.V. (2016) Mass spectrometry guided structural biology. Curr. Opin. Struct. Biol. 40, 136–144 10.1016/j.sbi.2016.09.008 27721169

[75] Gault J., Donlan J.A., Liko I., Hopper J.T., Gupta K., Housden N.G. et al. (2016) High-resolution mass spectrometry of small molecules bound to membrane proteins. Nat. Methods 13, 333–336 10.1038/nmeth.3771 26901650 PMC4856209

[76] Babel W., Wachter E., Aquila H. and Klingenberg M. (1981) Amino acid sequence determination of the ADP,ATP carrier from beef heart mitochondria. The sequence of the C-terminal acidolytic fragment. Biochim. Biophys. Acta 670, 176–180 10.1016/0005-2795(81)90006-4 6271240

[77] Klingenberg M. (1989) Molecular aspects of the adenine nucleotide carrier from mitochondria. Arch. Biochem. Biophys. 270, 1–14 10.1016/0003-9861(89)90001-5 2648994

[78] Walpole T.B., Palmer D.N., Jiang H., Ding S., Fearnley I.M. and Walker J.E. (2015) Conservation of complete trimethylation of lysine-43 in the rotor ring of c-subunits of metazoan adenosine triphosphate (ATP) synthases. Mol. Cell. Proteom. 14, 828–840 10.1074/mcp.M114.047456 PMC4390263 25608518

[79] Malecki J.M., Willemen H., Pinto R., Ho A.Y.Y., Moen A., Eijkelkamp N. et al. (2019) Human FAM173A is a mitochondrial lysine-specific methyltransferase that targets adenine nucleotide translocase and affects mitochondrial respiration. J. Biol. Chem. 294, 11654–11664 10.1074/jbc.RA119.009045 31213526 PMC6682728

[80] Smith V.R., Fearnley I.M. and Walker J.E. (2003) Altered chromatographic behaviour of mitochondrial ADP/ATP translocase induced by stabilization of the protein by binding of 6'-O-fluorescein-atractyloside. Biochem. J. 376, 757–763 10.1042/bj20030942 14498831 PMC1223817

[81] Chorev D.S. and Robinson C.V. (2019) Response to comment on “Protein assemblies ejected directly from native membranes yield complexes for mass spectrometry”. Science 366, eaax3102 10.1126/science.aax3102 31699906

[82] Liebschner D., Afonine P.V., Moriarty N.W., Poon B.K., Sobolev O.V., Terwilliger T.C. et al. (2017) Polder maps: improving OMIT maps by excluding bulk solvent. Acta Crystallogr. D Struct. Biol. 73, 148–157 10.1107/S2059798316018210 28177311 PMC5297918

[83] Mifsud J., Ravaud S., Krammer E.M., Chipot C., Kunji E.R.S., Pebay-Peyroula E. et al. (2013) The substrate specificity of the human ADP/ATP carrier AAC1. Mol. Membr. Biol. 30, 160–168 10.3109/09687688.2012.745175 23173940

[84] Wang Y. and Tajkhorshid E. (2008) Electrostatic funneling of substrate in mitochondrial inner membrane carriers. Proc. Natl. Acad. Sci. U.S.A. 105, 9598–9603 10.1073/pnas.0801786105 18621725 PMC2474497

[85] Dehez F., Pebay-Peyroula E. and Chipot C. (2008) Binding of ADP in the mitochondrial ADP/ATP carrier is driven by an electrostatic funnel. J. Am. Chem. Soc. 130, 12725–12733 10.1021/ja8033087 18729359

[86] Beyer K. and Klingenberg M. (1985) ADP/ATP carrier protein from beef heart mitochondria has high amounts of tightly bound cardiolipin, as revealed by 31P nuclear magnetic resonance. Biochemistry 24, 3821–3826 10.1021/bi00336a001 2996583

[87] Schlame M., Beyer K., Hayer-Hartl M. and Klingenberg M. (1991) Molecular species of cardiolipin in relation to other mitochondrial phospholipids. Is there an acyl specificity of the interaction between cardiolipin and the ADP/ATP carrier? Eur. J. Biochem. 199, 459–466 10.1111/j.1432-1033.1991.tb16144.x 1649052

[88] Haghighi A., Haack T.B., Atiq M., Mottaghi H., Haghighi-Kakhki H., Bashir R.A. et al. (2014) Sengers syndrome: six novel AGK mutations in seven new families and review of the phenotypic and mutational spectrum of 29 patients. Orphanet J. Rare Dis. 9, 119 10.1186/s13023-014-0119-3 25208612 PMC4167147

[89] Mayr J.A., Haack T.B., Graf E., Zimmermann F.A., Wieland T., Haberberger B. et al. (2012) Lack of the mitochondrial protein acylglycerol kinase causes Sengers syndrome. Am. J. Hum. Genet. 90, 314–320 10.1016/j.ajhg.2011.12.005 22284826 PMC3276657

[90] Jordens E.Z., Palmieri L., Huizing M., van den Heuvel L.P., Sengers R.C., Dorner A. et al. (2002) Adenine nucleotide translocator 1 deficiency associated with Sengers syndrome. Ann. Neurol. 52, 95–99 10.1002/ana.10214 12112053

[91] Crichton P.G., Lee Y., Ruprecht J.J., Cerson E., Thangaratnarajah C., King M.S. et al. (2015) Trends in thermostability provide information on the nature of substrate, inhibitor, and lipid interactions with mitochondrial carriers. J. Biol. Chem. 290, 8206–8217 10.1074/jbc.M114.616607 25653283 PMC4375477

[92] Kadenbach B., Mende P., Kolbe H.V., Stipani I. and Palmieri F. (1982) The mitochondrial phosphate carrier has an essential requirement for cardiolipin. FEBS Lett. 139, 109–112 10.1016/0014-5793(82)80498-5 7075760

[93] Hoffmann B., Stockl A., Schlame M., Beyer K. and Klingenberg M. (1994) The reconstituted ADP/ATP carrier activity has an absolute requirement for cardiolipin as shown in cysteine mutants. J. Biol. Chem. 269, 1940–1944 PMID: 8294444

[94] Duncan A.L., Ruprecht J.J., Kunji E.R.S. and Robinson A.J. (2018) Cardiolipin dynamics and binding to conserved residues in the mitochondrial ADP/ATP carrier. Biochim. Biophys. Acta 1860, 1035–1045 10.1016/j.bbamem.2018.01.017 PMC5988563 29366674

[95] Hedger G., Rouse S.L., Domanski J., Chavent M., Koldso H. and Sansom M.S. (2016) Lipid-loving ANTs: molecular simulations of cardiolipin interactions and the organization of the adenine nucleotide translocase in model mitochondrial membranes. Biochemistry 55, 6238–6249 10.1021/acs.biochem.6b00751 27786441 PMC5120876

[96] Dehez F., Schanda P., King M.S., Kunji E.R.S. and Chipot C. (2017) Mitochondrial ADP/ATP carrier in dodecylphosphocholine binds cardiolipins with non-native affinity. Biophys. J. 113, 2311–2315 10.1016/j.bpj.2017.09.019 29056231 PMC5722206

[97] Crichton P.G., Lee Y. and Kunji E.R.S. (2017) The molecular features of uncoupling protein 1 support a conventional mitochondrial carrier-like mechanism. Biochimie 134, 35–50 10.1016/j.biochi.2016.12.016 28057583 PMC5395090

[98] Harmel R. and Fiedler D. (2018) Features and regulation of non-enzymatic post-translational modifications. Nat. Chem. Biol. 14, 244–252 10.1038/nchembio.2575 29443975

[99] Garland P.B., Shepherd D. and Yates D.W. (1965) Steady-state concentrations of coenzyme A, acetyl-coenzyme A and long-chain fatty acyl-coenzyme A in rat-liver mitochondria oxidizing palmitate. Biochem. J. 97, 587–594 10.1042/bj0970587 16749169 PMC1264680

[100] Drazic A., Myklebust L.M., Ree R. and Arnesen T. (2016) The world of protein acetylation. Biochim. Biophys. Acta 1864, 1372–1401 10.1016/j.bbapap.2016.06.007 27296530

[101] Wagner G.R. and Payne R.M. (2013) Widespread and enzyme-independent Nε-acetylation and Nε-succinylation of proteins in the chemical conditions of the mitochondrial matrix. J. Biol. Chem. 288, 29036–29045 10.1074/jbc.M113.486753 23946487 PMC3790002

[102] Weinert B.T., Iesmantavicius V., Moustafa T., Scholz C., Wagner S.A., Magnes C. et al. (2014) Acetylation dynamics and stoichiometry in *Saccharomyces cerevisiae*. Mol. Syst. Biol. 10, 716 10.1002/msb.134766 24489116 PMC4023402

[103] Weinert B.T., Scholz C., Wagner S.A., Iesmantavicius V., Su D., Daniel J.A. et al. (2013) Lysine succinylation is a frequently occurring modification in prokaryotes and eukaryotes and extensively overlaps with acetylation. Cell Rep. 4, 842–851 10.1016/j.celrep.2013.07.024 23954790

[104] Britton L.M., Newhart A., Bhanu N.V., Sridharan R., Gonzales-Cope M., Plath K. et al. (2013) Initial characterization of histone H3 serine 10 O-acetylation. Epigenetics 8, 1101–1113 10.4161/epi.26025 23949383 PMC3891691

[105] Mukherjee S., Keitany G., Li Y., Wang Y., Ball H.L., Goldsmith E.J. et al. (2006) Yersinia YopJ acetylates and inhibits kinase activation by blocking phosphorylation. Science 312, 1211–1214 10.1126/science.1126867 16728640

[106] Chorev D.S., Tang H., Rouse S.L., Bolla J.R., von Kugelgen A., Baker L.A. et al. (2020) The use of sonicated lipid vesicles for mass spectrometry of membrane protein complexes. Nat. Protoc. 15, 1690–1706 10.1038/s41596-020-0303-y 32238951 PMC7305028

[107] Sanchez J.F., Kauffmann B., Grelard A., Sanchez C., Trezeguet V., Huc I. et al. (2012) Unambiguous structure of atractyloside and carboxyatractyloside. Bioorg. Med. Chem. Lett. 22, 2973 10.1016/j.bmcl.2012.02.040 22425567

